# Supplementary material for: Incoherent-mediator for quantum state transfer in the ultrastrong coupling regime
Source: Sci Rep. 2017 Jun 23;7:4157. doi: 10.1038/s41598-017-04467-1 (PMC5482826; doi:10.1038/s41598-017-04467-1)
Supplement: Supplementary file 1 — Supplemental material: Incoherent-mediator for quantum state transfer in the ultrastrong coupling regime [file 41598_2017_4467_MOESM1_ESM.pdf]

Supplemental material: Incoherent-mediator for quantum state transfer in the ultrastrong coupling regime

F. A. Cárdenas-López[1,2,†], F. Albarrán-Arriagada[1], G. Alvarado Barrios[1], J. C. Retamal[1,2,*], and G. Romero[1,+]

[1]Departamento de Física, Universidad de Santiago de Chile (USACH), Avenida Ecuador 3493, 9170124, Santiago, Chile
[2]Center for the Development of Nanoscience and Nanotechnology 9170124, Estación Central, Santiago, Chile

The derivation of the effective Hamiltonian

Here, we derive the effective Hamiltonian described in the main manuscript where a two-qubit quantum Rabi system interacts with two additional qubits. We write the QRS Hamiltonian in its diagonal form ($\hbar = 1$)

$$H_{\rm QRS} = \sum_{j=0}^{\infty} \nu_j |\psi_j\rangle\langle\psi_j|, \tag{1}$$

where ν_j is the jth eigenfrequency associated with the jth eigenstate $|\psi_j\rangle$. Also, we make use of the completeness relation for eigenstates $|\psi_j\rangle$ to write the Hamiltonian (2) of the main manuscript as

$$H = \sum_{j=0}^{\infty} \nu_j |\psi_j\rangle\langle\psi_j| + \sum_{n=1}^{N=2} \frac{\omega_n}{2}\tau_n^z + \sum_{n=1}^{N=2}\sum_{j,k} \lambda_n \chi_{jk} \tau_n^x |\psi_j\rangle\langle\psi_k|, \tag{2}$$

where we have defined $\chi_{jk} = \langle\psi_j|(a+a^\dagger)|\psi_k\rangle$. To derive the effective qubit-qubit Hamiltonian, we consider the Hamiltonian (2) in the interaction picture with respect to the free part $H_0 = \sum_{j=0}^{\infty}\nu_j|\psi_j\rangle\langle\psi_j| + \sum_{n=1}^{N=2}(\omega_n/2)\tau_n^z$. This leads to the Hamiltonian

$$\bar{H}_I(t) = \sum_{n,j,k} \lambda_n \chi_{jk} |\psi_j\rangle\langle\psi_k| \big(\tau_n^+ e^{i\Delta_{kj}^n t} + \tau_n^- e^{-i\mu_{kj}^n t}\big), \tag{3}$$

where the detuning parameters are defined as $\Delta_{kj}^n = \omega_n - \nu_{kj}$ and $\mu_{kj}^n = \omega_n + \nu_{kj}$, abd $\nu_{kj} = \nu_k - \nu_j$ are energy differences of the QRS. In addition, the effective Hamiltonian is given by

$$\bar{H}_{\rm eff}(t) = \frac{1}{2}[\bar{H}_I(t), W(t)], \tag{4}$$

where the function $W(t)$ is defined by the following integral

$$W(t) = -i\int_{t_0}^{t} \bar{H}_I(t')dt'. \tag{5}$$

From the Hamiltonian (3) it is straightforward to obtain $W(t)$ as

$$W(t) = \sum_{n=1}^{N=2}\sum_{j,k}\left(\lambda_n\chi_{jk}\tau_n^-|\psi_j\rangle\langle\psi_k|\frac{(e^{-i\mu_{kj}^n t}-1)}{\mu_{kj}^n} - \lambda_n\chi_{jk}\tau_n^+|\psi_j\rangle\langle\psi_k|\frac{(e^{i\Delta_{kj}^n t}-1)}{\Delta_{kj}^n}\right). \tag{6}$$

Hence, the derivation of the effective Hamiltonian (4) depends only on the commutator between terms (6) and (3). In this case, the most relevant commutator are given by

$$[\tau_n^{\pm}|\psi_j\rangle\langle\psi_k|, \tau_m^{\pm}|\psi_p\rangle\langle\psi_q|] = \tau_n^{\pm}\tau_m^{\pm}(\delta_{kp}|\psi_j\rangle\langle\psi_q| - \delta_{jq}|\psi_p\rangle\langle\psi_k|) \tag{7a}$$

$$[\tau_n^{\pm}|\psi_j\rangle\langle\psi_k|, \tau_m^{\mp}|\psi_p\rangle\langle\psi_q|] = \tau_n^{\pm}\tau_m^{\mp}\delta_{kp}|\psi_j\rangle\langle\psi_q| - \tau_m^{\mp}\tau_n^{\pm}\delta_{jq}|\psi_p\rangle\langle\psi_k|. \tag{7b}$$

Thus, the effective Hamiltonian reads

$$\begin{aligned}\bar{H}_{\rm eff}(t) = \frac{1}{2}\sum_{n,n'}\sum_{j,k}\lambda_n\lambda_{n'}\chi_{jk}\times\Bigg[\\
&\sum_p \chi_{pj}|\psi_p\rangle\langle\psi_k|\Bigg(\frac{e^{i\Delta_{kj}^n t}(e^{i\Delta_{jp}^{n'}t}-1)\tau_n^+\tau_{n'}^+}{\Delta_{jp}^{n'}} - \frac{e^{i\Delta_{kj}^n t}(e^{i\mu_{jp}^{n'}t}-1)\tau_{n'}^-\tau_n^+}{\mu_{jp}^{n'}}\\
&+\frac{e^{-i\mu_{kj}^n t}(e^{i\Delta_{jp}^{n'}t}-1)\tau_{n'}^+\tau_n^-}{\Delta_{jp}^{n'}} - \frac{e^{-i\mu_{kj}^n t}(e^{-i\mu_{jp}^{n'}t}-1)\tau_n^-\tau_{n'}^-}{\mu_{jp}^{n'}}\Bigg)\\
&-\sum_q \chi_{kq}|\psi_j\rangle\langle\psi_q|\Bigg(\frac{e^{i\Delta_{kj}^n t}(e^{i\Delta_{qk}^{n'}}t-1)\tau_n^+\tau_{n'}^+}{\Delta_{qk}^{n'}} - \frac{e^{i\Delta_{kj}^n t}(e^{i\mu_{qk}^{n'}t}-1)\tau_{n'}^+\tau_n^-}{\mu_{qk}^{n'}}\\
&+\frac{e^{-i\mu_{kj}^n t}(e^{i\Delta_{qk}^{n}t}-1)\tau_n^-\tau_{n'}^+}{\Delta_{qk}^{n'}} - \frac{e^{-i\mu_{kj}^n t}(e^{-i\mu_{qk}^{n'}t}-1)\tau_n^-\tau_{n'}^-}{\mu_{qk}^{n'}}\Bigg)\Bigg].\end{aligned} \tag{8}$$

It is noteworthy that in our system, the frequency terms μ_{kj}^n, Δ_{kj}^n and $\nu_{k,k'}$ are larger than the effective coupling terms $\lambda_n\lambda_{n'}\chi_{jk}\chi_{pj}/\Delta_{kj}^{n'}$ and $\lambda_n\lambda_{n'}\chi_{jk}\chi_{pj}/\mu_{kj}^{n'}$. Therefore, we can be safely neglect these fast oscillating terms in a secular approximation. In this case, the effective Hamiltonian in the Schrödinger picture reads

$$H_{\rm eff} = H_0 + \frac{1}{2}\sum_{n,n'}\sum_{j,k}\lambda_n\lambda_{n'}|\chi_{jk}|^2(|\psi_k\rangle\langle\psi_k| - |\psi_j\rangle\langle\psi_j|)\left(\frac{\tau_n^+\tau_{n'}^+}{\Delta_{jk}^{n'}} - \frac{\tau_n^+\tau_{n'}^-}{\mu_{jk}^{n'}} + \frac{\tau_n^-\tau_{n'}^+}{\Delta_{jk}^{n'}} - \frac{\tau_n^-\tau_{n'}^-}{\mu_{jk}^{n'}}\right). \tag{9}$$

In addition, as the effective Hamiltonian contains terms of the form $\tau_n^{\pm}\tau_{n'}^{\pm}$, that are zero when $n = n'$, we can split the sum into two parts, one for equal indexes n and n', and one for the opposite case. In this situation, we obtain an effective qubit-qubit interaction between the leftmost and rightmost qubits

$$H_{\rm eff} = H_0 + \frac{1}{2}\sum_{j,k}|\chi_{jk}|^2(|\psi_k\rangle\langle\psi_k| - |\psi_j\rangle\langle\psi_j|)\Bigg[\sum_n\lambda_n^2\left(\frac{\tau_n^-\tau_{n'}^+}{\Delta_{jk}^{n'}} - \frac{\tau_n^+\tau_{n'}^-}{\mu_{jk}^{n'}}\right) + \sum_{n,n'}\lambda_n\lambda_{n'}\left(\frac{1}{\Delta_{jk}^{n'}} + \frac{1}{\Delta_{jk}^{n'}} - \frac{1}{\mu_{jk}^{n'}} - \frac{1}{\mu_{jk}^{n'}}\right)\tau_n^x\tau_{n'}^x\Bigg]. \tag{10}$$

Finally, if we truncate to the two lowest energy levels of the QRS, we obtain the effective Hamiltonian

$$H_{\rm eff} = H_0 + \frac{1}{2}|\chi_{10}|^2\mathbf{Z}_p\otimes\mathbf{S}_{12}, \tag{11}$$

where $|\chi_{10}|^2 = |\langle\psi_0|(a^\dagger + a)|\psi_1\rangle|^2$, $\mathbf{Z}_p = |\psi_1\rangle\langle\psi_1| - |\psi_0\rangle\langle\psi_0|$, $\mathbf{S}_{12} = \lambda_1\lambda_2(1/\mu_{10}^1 + 1/\mu_{10}^2 - 1/\Delta_{10}^1 - 1/\Delta_{10}^2)\tau_1^x\tau_2^x + 2\sum_{n=1}^2\lambda_n^2(\tau_n^+\tau_n^-/\Delta_{10}^n - \tau_n^-\tau_n^+/\mu_{10}^n)$, and $\tau_n^{\pm} = (\tau_n^x \pm i\tau_n^y)/2$. The detunings are defined as $\Delta_{10}^n = \omega_n - \nu_{10}$ and $\mu_{10}^n = \omega_n + \nu_{10}$, where ω_n corresponds to the nth qubit frequency that interacts with the QRS.

Experimental Proposal

We propose an experimental realization of our quantum state transfer protocol based on a circuit QED architecture. The circuit for the implementation of our model is depicted in Figure (1), with a superconducting coplanar waveguide resonator (CPWR) of length L which supports charge-current waves with phase velocity $v = 1/\sqrt{lc}$ and impedance $Z_0 = \sqrt{l/c}$, where l and c are the inductance and capacitance per unit length. The CPWR is embedded by $N = 2$ identical Josephson junction (JJs). The JJs are characterized by their Josephson inductance L_J, capacitance C_J and plasma frequency $\omega_p = 1/\sqrt{C_J L_J}$. Moreover, two superconducting flux qubits formed by three Josephson junction are galvanically coupled to the CPWR through of the embedded junction, see Figure (1). This configuration allows to achieve ultrastrong coupling regime between the flux qubits and the resonator [1]. In addition, two transmon qubits [2] are capacitively coupled to the edges of the CPWR. In the circuir network theory [3, 4], the circuit is described in terms of the flux nodes $\phi(x,t) = \int_{-\infty}^{t} dt' V(x,t')$, where $V(x,t')$ is the voltage drop through the specific branch component. The circuit Lagrangian reads ($N = 2$)

$$\mathcal{L} = \sum_{i=1}^{N} \mathcal{L}_{\rm JJ}^{(i)} + \sum_{i=1}^{N+1} \mathcal{L}_{\rm Tl}^{(i)} + \sum_{j=1}^{2} \mathcal{L}_{\rm Q}^{(j)} + \mathcal{L}_{\rm I}, \tag{12}$$

where

$$\mathcal{L}_{\rm JJ}^{(i)} = \sum_{l=1}^{4} \frac{C_{J_l}^{(i)}}{2} (\dot{\phi}_l^{(i)})^2 + E_{J_l}^{(i)} \cos\left(\frac{\phi_l^{(i)}}{\varphi_0}\right) \tag{13}$$

$$\mathcal{L}_{\rm Tl}^{(i)} = \sum_{k=0}^{n} \frac{c \cdot \Delta x}{2} (\dot{\psi}_k^{(i)})^2 - \sum_{k=0}^{n-1} \frac{1}{2l \cdot \Delta x} (\psi_k^{(i)} - \psi_{k+1}^{(i)})^2 \tag{14}$$

$$\mathcal{L}_{\rm Q}^{(j)} = \frac{C_\tau^{(j)} + C_c}{2} (\dot{\phi}_\tau^{(j)})^2 + E_{\tau,j} \cos\left(\frac{\phi_\tau^{(j)}}{\varphi_0}\right) \tag{15}$$

$$\mathcal{L}_{\rm I} = \frac{C_c}{2} (\dot{\phi}_\tau^1 - \dot{\psi}_0^{(1)})^2 + \frac{C_c}{2} (\dot{\phi}_\tau^2 - \dot{\psi}_n^{(N+1)})^2. \tag{16}$$

Here $\psi_k^{(i)}$, $\phi_l^{(i)}$ and $\phi_\tau^{(j)}$ correspond to the flux variables that describe the CPWR, the Josephson junctions in each superconducting loop, and the transmon qubits, respectively. Furthermore, $C_{J_l}^{(i)}$ and $E_{J_l}^{(i)}$ are the Josephson capacitance and energy on each JJ that compound the flux qubit and the embedded junction. $C_\tau^{(j)}$ are the total capacitances of each transmon qubit and $E_{\tau,j}$ is their respective Josephson energy. C_c is the coupling capacitance between the transmon qubit and the CPWR, and Δx is the lattice spacing of the lumped circuit element description.

First, we consider the Josephson junction Lagrangian $\mathcal{L}_{\rm JJ}^{(i)}$ whose circuit is shown in Figure 1(b)

$$\mathcal{L}_{\rm JJ}^{(i)} = \sum_{l=1}^{4} \frac{C_{J_l}^{(i)}}{2} (\dot{\phi}_l^{(i)})^2 + E_{J_l}^{(i)} \cos\left(\frac{\phi_l^{(i)}}{\varphi_0}.\right) \tag{17}$$

The device $F^{(j)}$ is a superconducting loop intersected by four JJs. The Josephson junction's configuration give us a quantization for each closed loop i.e. $\sum_l \varphi_l^{(i)} = 2\pi n + 2\pi f_x^{(i)}$, where $\varphi_l^{(i)} = \phi_l^{(i)}/\Phi_0$ is the superconducting phase different along of the lth junction, $f_x^{(i)}$ is the frustration parameter defined as $f_x^{(i)} = \Phi_x^{(i)}/\Phi_0$, and $\Phi_0 = h/2e$ is the flux quantum. We assume $E_{J_1}^{(i)} = E_{J_1}^{(i)} = E_J^{(i)}$, $E_{J_3}^{(i)} = \alpha E_J^{(i)}$ and $E_{J_4}^{(i)} = \gamma E_J^{(i)}$. Moreover, $\delta\psi^{(i)}$ is the phase drop along the shared wire between the CPWR and the embedded Josephson junction. Taking into account the quantization rule for the superconducting loop, the $\mathcal{L}_{\rm JJ}^{(i)}$ takes the following form

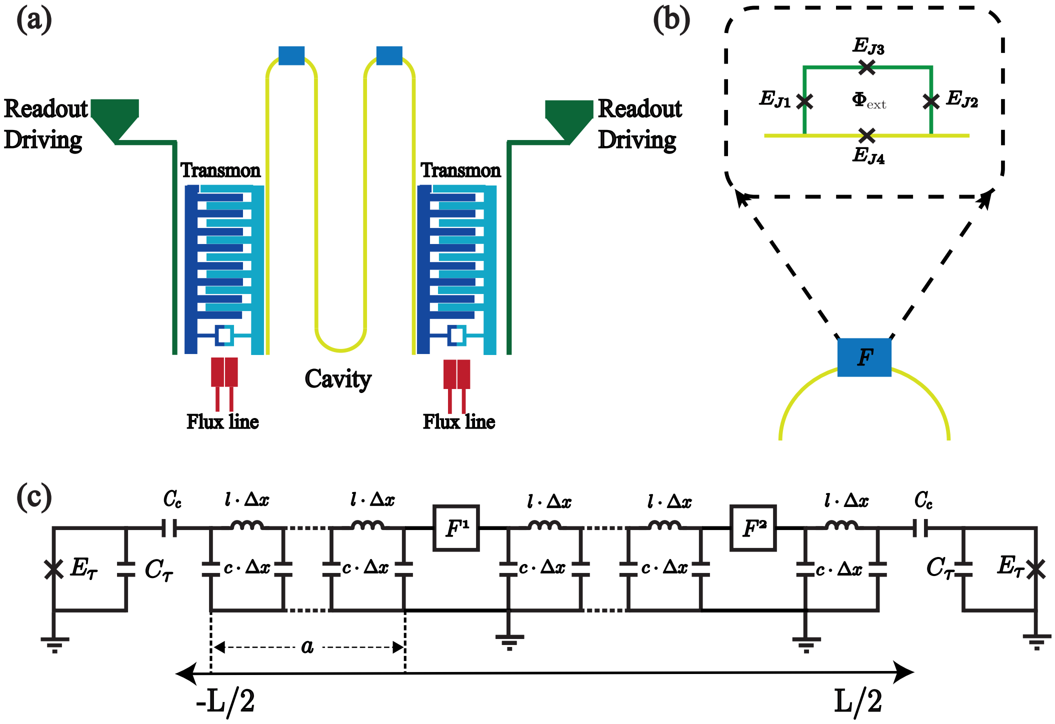

Figure 1: **Schematic of the experimental proposal.** (a) A superconducting $\lambda/2$ coplanar waveguide resonator is galvanically coupled to $N = 2$ superconducting loops formed by four Josephson junctions. Also, the resonator is coupled capacitively to two transmon devices at the edges of the waveguide. (b) Enlarged view of figure (a) at the position where the superconducting loop is placed. The flux qubit is formed by three Josephson junctions and the coupling between each flux qubit and the resonator is mediated by the the fourth embedded junction. (c) Equivalent circuit for (a), the resonator is considered as a finite set of LC circuits inductively connected in a series, these LC circuits are characterized by the capacitance $c \cdot \Delta x$ and inductance $l \cdot \Delta x$, the flux qubits are denoted by $F^{(j)}$ and the transmon devices are formed by two superconducting islands shunted by a large capacitance $C_\mathcal{T}$ and an SQUID loop.

$$
\begin{aligned}
\mathcal{L}_{\rm JJ}^{(i)} &= \frac{C_J^{(i)}}{2}\left[\dot{\phi}_1^{(i)2} + \dot{\phi}_2^{(i)2} + \alpha(\dot{\phi}_1^{(i)2} - \dot{\phi}_2^{(i)2} + \delta\dot{\psi}^{(i)} + 2\pi \dot{f_x}^{(i)})^2 + \gamma\delta\dot{\psi}^{(i)2}\right] \\
&+E_J^{(i)}\left[\cos\left(\frac{\phi_1^{(i)}}{\Phi_0}\right) + \cos\left(\frac{\phi_2^{(i)}}{\Phi_0}\right) + \alpha\cos\left(\frac{\phi_1^{(i)}}{\Phi_0} - \frac{\phi_1^{(i)}}{\Phi_0} + \frac{\delta\psi^{(i)}}{\Phi_0} + 2\pi f_x^{(i)}\right) + \gamma\cos\left(\frac{\delta\psi^{(i)}}{\Phi_0}\right)\right] \qquad (18)
\end{aligned}
$$

We consider that the external flux $f_x^{(i)}$ is static, therefore it does not present temporal variation $\dot{f}_x^{(i)} = 0$. Also, we work in the regime where the Josephson energy of the embedded junction is much larger than its charging energy. In this case, the phase $\delta\psi^{(i)}/\varphi_0$ is well localized allowing to expand the potential energy of (18) up to the lowest orders in $\delta\psi^{(i)}/\varphi_0$, since, in general, $\delta\psi^{(i)}/\varphi_0 \ll 1$ [8]. Then, $\mathcal{L}_{\rm JJ}^{(i)}$ reads

$$\mathcal{L}_{\rm JJ}^{(i)} = \frac{C_J^{(i)}}{2}\big[(1+\alpha)(\dot\phi_1^{(i)2}+\dot\phi_2^{(i)2}) - 2\alpha\dot\phi_1^{(i)2}\dot\phi_2^{(i)2} + 2\alpha\delta\dot\psi^{(i)}(\dot\phi_1^{(i)2}-\dot\phi_2^{(i)2}) + (\alpha+\gamma)(\delta\dot\psi^{(i)})^2\big]$$
$$+E_J^{(i)}\big[\cos\varphi_1^{(i)}+\cos\varphi_2^{(i)}+\alpha\cos(\varphi_1^{(i)}-\varphi_2^{(i)}+2\pi f_x^{(i)}) + \gamma\cos(\delta\psi^{(i)}/\Phi_0) - \alpha\sin(\varphi_1^{(i)}-\varphi_2^{(i)}+2\pi f_x^{(i)})\delta\psi^{(i)}\big]. \quad (19)$$

From the above expression, we can observe three important contributions terms

$$\mathcal{L}_{\rm JJ}^{(i)} = \mathcal{L}_{\rm flux}^{(i)} + \mathcal{L}_{\rm int}^{(i)} + \mathcal{L}_{\rm emb}^{(i)}$$

$$\mathcal{L}_{\rm flux}^{(i)} = \frac{C_J^{(i)}}{2}\big[(1+\alpha)(\dot\phi_1^{(i)2}+\dot\phi_2^{(i)2}) - 2\alpha\dot\phi_1^{(i)2}\dot\phi_2^{(i)2}\big] + E_J^{(i)}\big[\cos\varphi_1^{(i)}+\cos\varphi_2^{(i)}+\alpha\cos(\varphi_1^{(i)}-\varphi_2^{(i)}+2\pi f_x^{(i)})\big] \quad (20)$$

$$\mathcal{L}_{\rm int}^{(i)} = \frac{C_J^{(i)}}{2}\big[2\alpha\delta\dot\psi^{(i)}(\dot\phi_1^{(i)2}-\dot\phi_2^{(i)2}) - \alpha\sin(\varphi_1^{(i)}-\varphi_2^{(i)}+2\pi f_x^{(i)})\delta\psi^{(i)}\big] \quad (21)$$

$$\mathcal{L}_{\rm emb}^{(i)} = \frac{C_J^{(i)}}{2}\big[(\alpha+\gamma)(\delta\dot\psi^{(i)})^2\big] - \gamma E_J^{(i)}\cos(\delta\psi^{(i)}/\Phi_0) \quad (22)$$

The equation (20) corresponds to the flux qubit Lagrangian [6], $\mathcal{L}_{\rm int}^{(i)}$ stands for the capacitive and inductive interaction between the flux qubit and the CPWR, and the $\mathcal{L}_{\rm emb}^{(i)}$ is the embedded Josephson junction Lagrangian. In the charge basis, the flux qubit Hamiltonian (20) is expressed as

$$H_{\rm flux}^{(i)} = \frac{4E_C^{(i)}}{1+2\alpha}\left[(1+\alpha)(N_1^{(i)2}+N_2^{(i)2}) + 2\alpha N_1^{(i)}N_2^{(i)}\right] - E_J^{(i)}\big[\cos\varphi_1^{(i)}+\cos\varphi_2^{(i)}+\alpha\cos(\varphi_1^{(i)}-\varphi_2^{(i)}+2\pi f_x^{(i)})\big]. \quad (23)$$

Where $E_C^{(i)} = e^2/2C_J^{(i)}$ and $N_j^{(i)} = -Q_j^{(i)}/2e$, with $Q_j^{(i)}$ the canonical momenta of $\phi_j^{(i)}$. $Q_j^{(i)}$ represents the charge on the flux qubit circuit. Likewise, the interacting Hamiltonian derived from $\mathcal{L}_{\rm int}^{(i)}$ in the charge basis reads

$$H_{\rm int}^{(i)} = -\frac{8\alpha C_J^{(i)}E_C^{(i)}}{1+2\alpha}\big(N_1^{(i)}-N_2^{(2)}\big)\delta\dot\psi^{(i)} + \alpha E_J^{(i)}\sin(\varphi_1^{(i)}-\varphi_2^{(i)}+2\pi f_x^{(i)})\delta\psi^{(i)}. \quad (24)$$

We consider that the presence of the flux qubit does not affect much the mode distribution of the CPWR. This can be achieved by considering the inductance on each piece of shared wire between the CPWR and the embedded JJ is much smaller than the total inductance of the flux qubit. In this situation, most of current will flow through the CPWR and we can treat the CPWR and the flux qubits as separated entities. Furthermore, it as been proven that the capacitive coupling in (24) is negligible as compared with the inductive coupling by several order of magnitudes due to very small Josephson capacitances [1]. In this case, the interaction Hamiltonian can be approximated by

$$H_{\rm int}^{(i)} = \alpha E_J^{(i)}\sin(\varphi_1^{(i)}-\varphi_2^{(i)}+2\pi f_x^{(i)})\delta\psi^{(i)}. \quad (25)$$

Close to the symmetry point $f_x^{(i)} = \Phi_0/2$, as shown in Figure 2(a), the flux qubit Hamiltonian (23) can be truncated up to its two lowest energy levels leading an effective two-level system

$$H_{\rm flux}^{(i)} = \frac{\omega_{q,i}}{2}\sigma_i^z \quad (26)$$

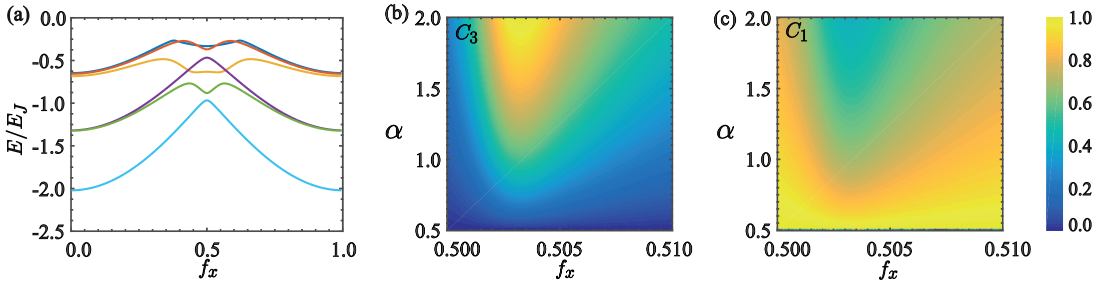

Figure 2: **Flux qubit spectrum.** (a) Energy spectrum of the flux qubit Hamiltonian (23) in the charge basis as a function of the frustration parameter $f_x^{(i)}$ and $\alpha = 0.75$. Close to the symmetry point, $f_x^{(i)} = \Phi_0/2$, the spectrum behaves as an effective two level system. (b-c) coupling coefficients $C_{1,3}$ as a function of α and $f_x^{(i)}$ in the vicinity of the symmetry point coefficient C_1 becomes maximal.

where $\omega_{q,i} = \sqrt{\Delta^2+\epsilon^2}$, being Δ the gap of the level crossing and $\epsilon = 2I_p(\Phi_x^{(i)} - \Phi_0/2)$ with I_p the persistent current. In the two-level system basis, numerical diagonalization shows that the qubit-resonator interacting term $\sin(\varphi_1^{(i)} - \varphi_2^{(i)} + 2\pi f_x^{(i)})$ can be expressed as

$$H_{\text{int}}^{(i)} = \alpha E_J^{(i)} \sin(\varphi_1^{(i)} - \varphi_2^{(i)} + 2\pi f_x^{(i)})\delta\psi^{(i)} = \alpha E_J^{(i)} \sum_{\rho=0,x,y,z} C_\rho^{(i)} \sigma_i^\rho \delta\psi^{(i)} \tag{27}$$

with $\sigma_i^0 = \mathbb{I}$ the identity matrix and C_ρ are parameters obtained numerically [8]. Numerical evidence shows that for the symmetry point $f_x = \Phi_0/2$ the coefficients C_ρ takes the following values $C_1 = 1$, $C_2 = C_0 = C_3 = 0$, see Fig 2(b) and Fig 2(c). In this case, the interacting qubit-resonator Hamiltonian reads ($\hbar = 1$)

$$H_{\text{int}}^{(i)} = \sigma_i^x \delta\psi^{(i)}. \tag{28}$$

For the external qubits we consider the Lagrangian for transmons [2] coupled at the ends of the CPWR

$$\mathcal{L}_\tau^{(j)} = \frac{C_\Sigma}{2}\phi_\tau^{(j)} + E_{\tau,j}\cos\left(\frac{\phi_\tau^{(j)}}{\Phi_0}\right) - C_c\phi_\tau^{(j)}\dot{\psi}(x,t), \tag{29}$$

where $C_\Sigma^{(j)} = C_c + C_\tau^{(j)}$ is the effective capacitance of each transmon, $\dot{\psi}(x,t)$ is proportional to the CPWR voltage evaluated at the end of the resonator i.e. $x = 0$ or $x = L$. The Hamiltonian is obtained by considering the canonical variable associated with the flux variable of the transmon. The quantization of the charge $N_{\tau,j} = -Q_{\tau,j}/2e$ leads to the following Hamiltonian

$$H_Q^{(j)} = H_\tau^{(j)} + H_{\text{int}}^{(j)}, \tag{30}$$

where

$$H_\tau^{(j)} = 4E_{C,j}N_{\tau,j}^2 - E_{\tau,j}\cos\left(\varphi_\tau^{(j)}\right) \tag{31}$$

$$H_{\text{int}}^{(j)} = E_{\text{cou}}N_{\tau,j}\dot{\psi}(x,t). \tag{32}$$

Here, $E_{C,j} = 2eC_c/C_\Sigma^{(j)}$ is the charging energy and $E_{\text{cou}} = 2eC_c/C_\Sigma^{(j)}$ is the coupling energy between the external qubits and the CPWR. In the transmon regime where $E_C \ll E_J$, it is possible to truncate up to the third level in

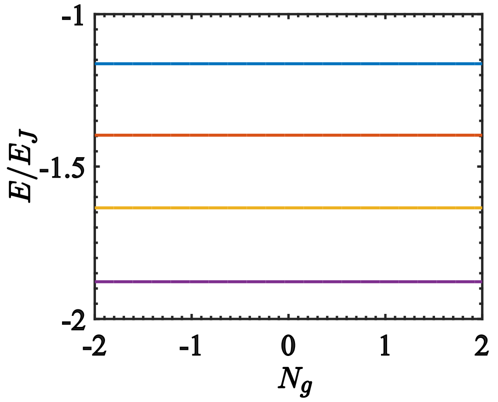

Figure 3: **Transmon energy spectrum.** Energy spectrum of the Hamiltonian (33) as a function of the parameter N_g for $E_{\tau,j} = 50E_{C,j}$. The anhamonicity for the three lower energy levels at $N_g = 0$ is $\alpha = E_{1,0}/E_J - E_{2,1}/E_J = 0.0039$, leading to a relative anharmonicity of about $\alpha/(E_{10}/E_J) \sim 1\%$. As the anhamonicity is very low the transmon needs to be treated as a effective three-level system.

the transmon energy spectrum, see Figure (3). In this case, the transmon Hamiltonian and its interaction with the resonator reads

$$
\begin{aligned}
H_{\tau}^{(j)} &= \sum_{n} \left[4E_{C,j}(n_j - N_g)^2 |n_j\rangle\langle n_j| + \frac{E_{\tau,j}}{2} \big(|n_j\rangle\langle n_j + 1| + |n_j + 1\rangle\langle n_j| \big) \right] && (33) \\
H_{\text{int}}^{(j)} &= E_{\text{cou}} \dot{\psi}(x,t) \sum_{n} |n_j\rangle\langle n_j|, && (34)
\end{aligned}
$$

with $N_g = V_g C_{\Sigma}^{(j)}/2e$, and V_g the gate voltage.

Finally, we derive the Hamiltonian and spectrum of a single transmission line resonator with $N = 2$ Josephson junctions embedded on it. We divide the CPWR in finite lump elements each represented as an LC resonator with capacitance $c \cdot \Delta x$ and inductance $l \cdot \Delta x$ as shown in Figure 1(b). The embedded Josephson junction are placed between the fluxes $\psi_n^{(i)}$ and $\psi_0^{(i+1)}$. The Lagrangian of the discretized CPWR reads

$$
\begin{aligned}
\mathcal{L}_{\text{CPWR}}^{(i)} &= \sum_{k=0}^{n} \frac{c \cdot \Delta x}{2} (\dot{\psi}_k^{(i)})^2 - \sum_{k=0}^{n-1} \frac{1}{2l \cdot \Delta x} (\psi_k^{(i)} - \psi_{k+1}^{(i)})^2 + \frac{C_J^{(i)}}{2} \left[(\alpha + \gamma)(\delta\dot{\psi}^{(i)})^2 \right] + \gamma E_J^{(i)} \cos(\delta\psi^{(i)}/\Phi_0) \\
&+ \frac{C_c}{2} \big((\dot{\psi_0}^{(1)})^2 + (\dot{\psi_n}^{(N+1)})^2 \big). && (35)
\end{aligned}
$$

To obtain the equations of motion of the CPWR we proceed similarly as done in Ref. [5]. We divide the Lagrangian of the embedded JJs in the linear $\mathcal{L}_{\text{JJ}}^{lin\ (i)}$ and non-linear part $\mathcal{L}_{\text{JJ}}^{nonlin\ (i)}$

$$
\begin{aligned}
\mathcal{L}_{\text{JJ}}^{lin\ (i)} &= \frac{C_J^{(i)}}{2} \left[(\alpha + \gamma)(\delta\dot{\psi}^{(i)})^2 \right] - \frac{1}{2L_J^{(i)}} \big(\delta\psi^{(i)} \big)^2 && (36) \\
\mathcal{L}_{\text{JJ}}^{nonlin\ (i)} &= \gamma E_J^{(i)} \cos(\delta\psi^{(i)}/\Phi_0) + \frac{1}{2L_J^{(i)}} \big(\delta\psi^{(i)} \big)^2. && (37)
\end{aligned}
$$

The linear part of the CPWR Lagrangian leads to a set of equations of motion at the ends and at the bulk of the CPWR

$$\big(c\cdot\Delta x + C_c\big)\ddot{\psi}_0^{(1)} = \frac{-1}{l\cdot\Delta x}\big(\psi_0^{(1)} - \psi_1^{(i)}\big)|_{x=0} \tag{38}$$

$$\big(c\cdot\Delta x + C_c\big)\ddot{\psi}_n^{(N+1)} = \frac{1}{l\cdot\Delta x}\big(\psi_{n-1}^{(N+1)} - \psi_n^{(N+1)}\big)|_{x=L} \tag{39}$$

$$c\cdot\Delta x\ \ddot{\psi}_q^{(1)} = \frac{1}{l\cdot\Delta x}\big(2\psi_q^{(i)} - \psi_{q+1}^{(i)} - \psi_{q-1}^{(i)}\big). \tag{40}$$

For the node fluxes at the embedded Josephson junction we need to consider that the JJ introduces a flux drop defined as $\delta\psi^{(i)} = \psi_n^{(i)} - \psi_0^{(i+1)}$, which corresponds to the flux approaching the JJ from the left $\psi_n^{(i)}$ and from the right $\psi_0^{(i+1)}$. The equation of motion for the JJs flux nodes are

$$c\cdot\Delta x\ddot{\psi}_n^{(i)} + (\alpha+\gamma)(\ddot{\psi}_n^{(i)} - \ddot{\psi}_0^{(i+1)}) = \frac{-1}{l\cdot\Delta x}\big(\psi_{n-1}^{(i)} - \psi_n^{(i)}\big) - \frac{1}{L_J^{(i)}}\big(\psi_n^{(i)} - \psi_0^{(i+1)}\big). \tag{41}$$

In the continuum limit ($\Delta x \to 0$) and under the condition $C_c \ll cL$, we obtain that the current flowing at the resonator edges must vanish, see Eq. 38 and Eq. 39. These boundary conditions together with the relation (41) lead a well defined eigenvalue problem [5] described by the set of equations

$$\partial_t^2\psi(x,t) = v^2\partial_x^2\psi(x,t) \tag{42}$$

$$\frac{1}{l}\partial_x\psi(x,t)|_{x=0} = 0 \qquad \frac{1}{l}\partial_x\psi(x,t)|_{x=L} = 0 \tag{43}$$

$$-\partial_x\psi(x=ia,t) = l(\alpha+\gamma)C_J^{(i)}\delta\ddot{\psi}^{(i)} - \frac{l}{L_J^{(i)}}\delta\psi^{(i)}. \tag{44}$$

As the embedded JJs in the CPWR only affect the spatial distribution in the flux distribution for the resonator, we can rewrite the expression (44) to obtain

$$-\partial_x\psi(x=ia,t) = \frac{l}{L_J^{(i)}}\left(1 - \frac{(\alpha+\gamma)\omega_r^2}{\omega_p^2}\right)\delta\psi^{(i)}, \tag{45}$$

where $a = L/(N+1)$ is the spacing between the embedded JJs.

Now we follow the procedure developed in references [7, 5], where the matrix transfer technique relates the spatial function of the flux at the ends of each CPWR. Also, we consider the flux distribution as $\psi(x,t) = \sum_r g_r(t)f_r(x)$ where $f_r(x) = A_r(x) + B_r(x)$. In this case, the solution wave equation leads to a set of orthonormal functions $f_r(x)$ that allow us to define the Hamiltonian

$$H_{\text{CPWR}} = \sum_r \frac{1}{2\eta_r}\pi_r^2 + \frac{1}{2}\eta_r\omega_r^2 g_r^2 + H_{\text{nonlin}}. \tag{46}$$

Here, $\pi_r = \eta_r\dot{g}_r$ is the canonical conjugate momenta of g_r, $\eta_r = \int_0^L \big(cf_r^2dx + \sum_{i=1}^{N+1}(\alpha+\gamma)C_J^{(i)}[\delta f_r]^2\big)$ is the effective mass of the rth eigenmode, and ω_r is the frequency associated with the rth eigenmode. The latter is calculated through the following transcendental equation

$$\frac{\cos\left(\frac{\omega_r a}{v}\right) - \cos(n\pi/(N+1))}{\sin\left(\frac{\omega_r a}{v}\right)} = \frac{1}{2C_JZ_0}\frac{\omega_r}{(\omega_p^2 - (\alpha+\gamma)\omega_r^2)} \tag{47}$$

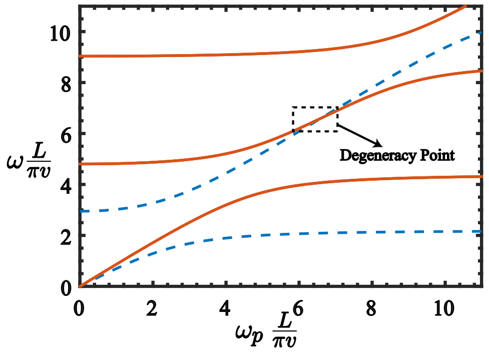

Figure 4: **Energy spectrum of the CPWR.** Energy spectrum of the coplanar waveguide resonator with two Josephson junctions embedded on it, as a function of the plasma frequency ω_p. The parameters for the resonator are $v = 0.98 \times 10^8$m/s, $Z_0 = 50\Omega$, $L = 0.28$mm, and the Josephson capacitance of the embedded junction is $C_{J_4} = 1$pF. For particular values of the plasma frequency, the eigenmodes belonging to an specific manifold become degenerate.

being $Z_0 = \sqrt{l/c}$ the wave-impedance. The manifold for each eigenmode of the resonator is shown in Figure (4). These considerations allow us to calculate $H_{\rm nonlin}$ as

$$H_{\rm nonlin} = -\sum_{i=1}^{N} \gamma E_J^{(i)} \cos(\delta\psi^{(i)}/\Phi_0) + \frac{1}{2L_J^{(i)}} \big(\delta\psi^{(i)}\big)^2. \tag{48}$$

The Hamiltonian (46) is quantized by introducing lowering and raising operators defined as $a_r = \sqrt{\frac{\eta_r\omega_r}{2}}\big(g_r + i\pi_r/\eta_r\omega_r\big)$ for each eigenmode of the manifold. Thus we arrive to the following Hamiltonian

$$H_{\rm CPWR} = \sum_{r\in\mathcal{M}} \omega_r a_r^\dagger a_r + H_{\rm nonlin}. \tag{49}$$

Furthermore, by using the definition of the operator a_r we can rewrite the drop flux as

$$\delta\psi^{(i)} = \sqrt{\frac{2}{N+1}} \sin\big(\frac{\pi i}{N+1}\big) \lambda(\omega_r)(a_r + a_r^\dagger) \tag{50}$$

$$\partial_t\psi(x,t) = V(x,t) = \sum_r \sqrt{\frac{\omega_r}{2\eta_r}} f_r (a_r + a_r^\dagger), \tag{51}$$

where $\lambda(\omega_r)$ is described in terms of the system parameters [5]. Then, the Hamiltonian for the complete circuit can be written as follows

$$H = \sum_{r\in\mathcal{M}} \omega_r a_r^\dagger a_r + \sum_i \frac{\omega_{q,i}}{2}\sigma_i^z + \sum_{r\in\mathcal{M},i} g_{r,i}\sigma_i^x (a_r + a_r^\dagger) \tag{52}$$

$$+ \sum_j \Bigg(\sum_n \Bigg[4E_{C,j}(n_j - N_g)^2 |n_j\rangle\langle n_j| + \frac{E_{\tau,j}}{2} \big(|n_j\rangle\langle n_j + 1| + |n_j + 1\rangle\langle n_j|\big) \Bigg] \Bigg) \tag{53}$$

$$+ \sum_{r\in\mathcal{M},j} \lambda_{j,r} |n_j\rangle\langle n_j| (a_r + a_r^\dagger) + H_{\rm nonlin}. \tag{54}$$

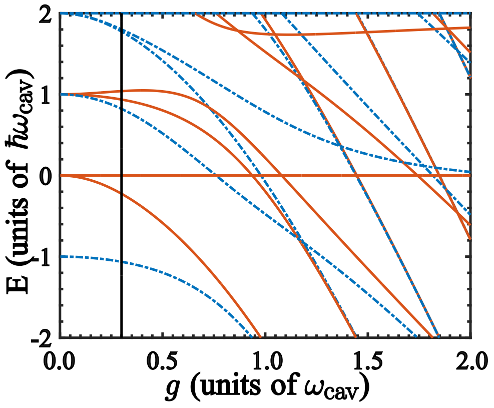

Figure 5: **Energy spectrum of the generalize Dicke model.** Energy spectrum of the Hamiltonian (58) with parameters $\omega_{q,1} = \omega_{q,2} = \omega_{\text{cav}}$, as a function of the coupling strength g. Blue (dot-dashed) lines stand for states with parity $p = +1$, and red (continuous) lines stand for states with parity $p = -1$. Straight lines stand for dark states.

Here, the coefficients $g_{r,i} = \sqrt{\frac{2}{N+1}} \sin\left(\frac{\pi i}{N+1}\right)\lambda(\omega_r)$ and $\lambda_{j,r} = E_{\text{cou}}\sqrt{\frac{\omega_r}{2\eta_r}} f_r$ depend on the system parameters. We focus on the situation where the plasma frequency takes an specific value $\omega_p = \pi v(N+1)/L$. In this case, the eigenmode frequency of the CPWR becomes degenerate [5] as shown in Figure (4). In addition, we also consider that the fourth Josephson junction works in the linearized regime (44) leading to $H_{\text{nonlin}} \approx 0$. Hence, the whole Hamiltonian reads

$$H = \sum_{r\in\mathcal{M}} \omega_r a_r^\dagger a_r + \sum_i \frac{\omega_{q,i}}{2}\sigma_i^z + \sum_{r\in\mathcal{M},i} g_{r,i}\sigma_i^x(a_r + a_r^\dagger) \tag{55}$$

$$+ \sum_j \left(\sum_n \left[4E_C^{(j)}(n_j - N_g)^2 |n_j\rangle\langle n_j| + \frac{E_{\tau,j}}{2}\left(|n_j\rangle\langle n_j+1| + |n_j+1\rangle\langle n_j|\right) \right] \right) \tag{56}$$

$$+ \sum_{r\in\mathcal{M},j} \lambda_{j,r}|n_j\rangle\langle n_j|(a_r + a_r^\dagger). \tag{57}$$

On the other hand, to study the dynamics of the system described by the above Hamiltonian, and how the third level of each the trasmon affect the process itself, let us consider that the frequency gap between the ground and first excited state of each transmon is resonant with the forbidden energy transition of the generalize Dicke model, that is, $E_{10} = \nu_{30}$. To be specific, see the vertical black line in Fig 5, where the forbidden transition corresponds to two consecutive blue (dot-dashed) lines. Similar to the study presented in the main manuscript, we consider the dynamics of excitation transfer between transmons. Numerical evidence for the generalized Dicke model and for the initial condition $|\psi_0\rangle = |1_1\rangle \otimes |0\rangle_{\text{QRS}} \otimes |0_2\rangle$ shows that the third energy level does not take part in the transfer process, see Figure 6 where we plot the states population of the transmon level as a function of the effective coupling strength calculated from the effective Hamiltonian (11). We see that the generalized Dickel model leads to a faster excitation transfer. Regarding the above results we estimate that the third energy level of each transmon can be safely neglected, leading an effective two-level system. In this case, the effective Hamiltonian of the whole systems depicted in Figure

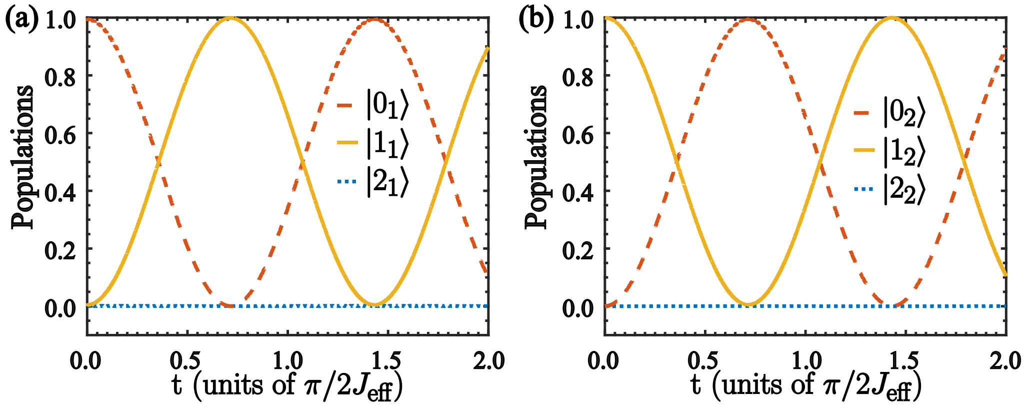

Figure 6: **Population evolution.** Population inversion of the transmon states for (a) the leftmost and (b) the rigthmost transmon. Numerical evidence shows that the third level on each transmon does not participate in the transfer process.

1 is described by the generalized Dicke model

$$H = H'_{\text{QRS}} + \sum_{r\in\mathcal{M},j} \frac{\omega_j}{2}\tau_j^z + \lambda_{j,r}\tau_j^x(a_r^\dagger + a_r) \tag{58}$$

$$H'_{\text{QRS}} = \sum_{r\in\mathcal{M}} \omega_r a_r^\dagger a_r + \sum_i \frac{\omega_{q,i}}{2}\sigma_i^z + \sum_{r\in\mathcal{M},i} g_{r,i}\sigma_i^x(a_r + a_r^\dagger). \tag{59}$$

References

[1] Bourassa, J. *et. al.* Ultrastrong coupling regime of cavity QED with phase-biased flux qubits. *Phys. Rev. A.* **80**, 032109 (2009).

[2] Koch, J. *et. al.* Charge-insensitive qubit design derived from the Cooper pair box. *Phys. Rev. A.* **76**, 042319 (2007).

[3] Yurke, B. & Denker, J. S. Quantum network theory. *Phys. Rev. A.* **29**, 1419 (1984).

[4] Devoret, M. H. Quantum Fluctuations in electrical circuits. *Les Houches Session LXIII. Ed. by S. Reynaud, E. Giacobino and J. Zinn-Justin.* 351 (1997).

[5] Leib, M., Hartmann, M. J. Synchronized switching in a Josephson junction crystal *Phys. Rev. Lett.* **112**, 223603 (2014).

[6] Orlando, T. P. *et. al.* Superconducting persistent-current qubit *Phys. Rev. B* **60**, 15398 (1999).

[7] Zueco, D., Mazo, J. J., Solano, E. & Ripoll, J. J. Microwave photonics with Josephson junction arrays: Negative refraction index and entanglement through disorder *Phys. Rev. B* **86**, 024503 (2012).

[8] Romero, G., Ballester, D., Wang, Y. M., Scarani, V. & Solano, E. Ultrafast Quantum Gates in Circuit QED *Phys. Rev. Lett* **108**, 120501 (2012).
